# Performance of a Four-Antigen *Staphylococcus aureus* Vaccine in Preclinical Models of Invasive *S. aureus* Disease

**DOI:** 10.3390/microorganisms9010177

**Published:** 2021-01-15

**Authors:** Ingrid L. Scully, Yekaterina Timofeyeva, Arthur Illenberger, Peimin Lu, Paul A. Liberator, Kathrin U. Jansen, Annaliesa S. Anderson

**Affiliations:** Pfizer Vaccine Research & Development, Pearl River, NY 10965, USA; ingrid.scully@pfizer.com (I.L.S.); yekaterina.timofeyeva@pfizer.com (Y.T.); arthur.illenberger@pfizer.com (A.I.); peiminlu@msn.com (P.L.); paul.liberator@pfizer.com (P.A.L.); kathrin.jansen@pfizer.com (K.U.J.)

**Keywords:** *Staphylococcus aureus*, invasive disease, surgery-associated infection, sepsis, SA4Ag vaccine, conjugated polysaccharide, ClfA, MntC, protection, animal models

## Abstract

A *Staphylococcus aureus* four-antigen vaccine (SA4Ag) was designed for the prevention of invasive disease in surgical patients. The vaccine is composed of capsular polysaccharide type 5 and type 8 CRM_197_ conjugates, a clumping factor A mutant (Y338A-ClfA) and manganese transporter subunit C (MntC). *S. aureus* pathogenicity is characterized by an ability to rapidly adapt to the host environment during infection, which can progress from a local infection to sepsis and invasion of distant organs. To test the protective capacity of the SA4Ag vaccine against progressive disease stages of an invasive *S. aureus* infection, a deep tissue infection mouse model, a bacteremia mouse model, a pyelonephritis model, and a rat model of infectious endocarditis were utilized. SA4Ag vaccination significantly reduced the bacterial burden in deep tissue infection, in bacteremia, and in the pyelonephritis model. Complete prevention of infection was demonstrated in a clinically relevant endocarditis model. Unfortunately, these positive preclinical findings with SA4Ag did not prove the clinical utility of SA4Ag in the prevention of surgery-associated invasive *S. aureus* infection.

## 1. Introduction

*Staphylococcus aureus* is a Gram-positive bacterium that is carried asymptomatically in the nares of 20–50% of the general population [1]. However, upon a breach of skin or mucosal barriers, it can cause a wide spectrum of diseases, ranging from relatively mild skin infections, such as carbuncles, to life-threatening wound and bloodstream infections [2]. *S. aureus* infections following surgery carry particularly high mortality rates, and survivors require an additional 13–17 days in the hospital, significantly increasing healthcare costs [3]. The burden of *S. aureus* disease is exacerbated by the prevalence of antibiotic-resistant *S. aureus* isolates [4], highlighting the need for an effective prophylactic vaccine.

A consideration in both development and preclinical evaluation of a *S. aureus* vaccine is the organism’s ability to rapidly adapt to the host microenvironment [5]. *S. aureus* can enter normally sterile sites through lesions such as those created during surgery or traumatic injury and rapidly deploy an array of pathogenesis mechanisms, rendering *S. aureus* a challenging vaccine target. Consequently, a licensed vaccine against *S. aureus* disease is not yet available, and a clinically validated correlate of protection has not yet been established. Prevention strategies for patients at high risk for invasive *S. aureus* disease, such as surgical patients, include decolonization and antibiotic prophylaxis [6]. These procedures are limited by the variable effectiveness of decolonization and by the development of antibiotic resistance [7,8,9]. Thus, alternative preventative strategies, such as prophylactic vaccines, would be a welcome addition to the clinician’s armamentarium.

With that aim, a four-antigen *S. aureus* vaccine (SA4Ag) was designed for the prevention of invasive *S. aureus* infections following elective surgery. Each vaccine component was carefully selected so that, when combined, the vaccine would contravene major *S. aureus* pathogenesis mechanisms, namely initial adhesion events (ClfA), nutrient acquisition sustaining growth (MntC), and evasion of neutrophil-mediated killing (MntC and capsular polysaccharide (CP) type 5 and type 8 conjugates). The adhesin ClfA enables the attachment of *S. aureus* to human fibrinogen, and antibodies directed against this protein inhibit ClfA-mediated fibrinogen binding [10]. MntC is a highly conserved component of the manganese transporter MntABC that is quickly upregulated in vivo [11] and is expressed during biofilm formation in animal models [12]. Manganese acquisition by *S. aureus* is important for both growth and evasion of neutrophil killing through detoxifying oxygen radicals [11,13]. Finally, the vaccine contains capsular polysaccharide type 5 and type 8 conjugated to cross-reactive material 197 (CRM_197_), which induce functional antibodies that kill the bacteria following opsonophagocytosis [14,15], the process whereby antibodies coat the bacterium, fix complement, and induce uptake and killing by phagocytes, such as neutrophils. It has become clear that antibodies that bind to a bacterial antigen, such as those measured by an enzyme-linked immunosorbent assay, are not always functional, especially in adults with pre-existing exposure to a pathogen. Thus, demonstration of functional antibody activity, such as through an opsonophagocytic killing assay or fibrinogen binding assay is important as another step in the evaluation of a vaccine candidate, along with in vivo proof of concept studies.

The correlate of protection for *S. aureus* is not yet known, and a vaccine for the prevention of *S. aureus* disease has yet to be commercially licensed. The two candidates previously advanced to the clinic were supported by preclinical serology [16] and/or murine sepsis model studies [17,18]. These studies demonstrated that the vaccine candidates were immunogenic in preclinical models, an important first step in selecting candidates to advance to the clinic. In the case of iron-regulated surface determinant B (IsdB), a single challenge model, the murine sepsis model, was used to support preclinical efficacy [17,18]. The murine sepsis model mimics hematogenous spread through the body, one important aspect of *S. aureus* infection. However, *S. aureus* has notoriously complex pathogenesis mechanisms, which can only be modeled using multiple preclinical models. The development of relevant preclinical models for *S. aureus* is challenging, as *S. aureus* is adapted to the human host environment, and preclinical models have failed to predict clinical efficacy [19,20]. Here, the ability of SA4Ag to protect against invasive disease is demonstrated in three preclinical rodent models of *S. aureus* infection, each mimicking a distinct phase of *S. aureus* infection, namely deep tissue invasion, bacteremia, and distal infection. In the absence of a defined correlate of protection, and due to the limited ability of any single preclinical model to predict *S. aureus* vaccine clinical success, demonstrating efficacy in multiple models of *S. aureus* invasive disease is relevant for advancing a vaccine into clinical trials.

## 2. Materials and Methods

### 2.1. Bacterial Strains

The *S. aureus* clinical isolates from Pfizer’s internal collection that were used for in vivo analyses are listed in Table 1. These strains represent a diverse set of clinical isolates.

### 2.2. Animal Studies

All animal work was performed in strict accordance with approved Institutional Animal Care and Use Committee protocols at an Association for Assessment and Accreditation of Laboratory Animal Care, International-accredited facility, under the following Animal Use Protocols: PRL-2011-00105 (approved in 2007), PRL-2011-00249 (approved in 2008), PRL-2011-00102 (approved in 2002) and PRL-2011-00338 (approved in 2002) for mouse studies, and PRL-2011-00440 (approved in 2002) for rat studies. For all animal studies, statistical significance was determined via Student’s *t*-test using Welch’s correction, and a *p* value of ≤ 0.05 was considered significant (GraphPad Prism). For each model, bacterial challenge dose and immunization schedule were optimized.

#### 2.2.1. Surgical Site Infection Mouse Model

CD-1 female mice (6–8–week–old, 10–20/group; Charles River Laboratories) were vaccinated subcutaneously at weeks 0, 3, and 6 with 100 µL volume containing 1 µg CP8-CRM_197_ + 1 µg CP5-CRM_197_ + 10 µg Y338A ClfA + 10 µg MntC (SA4Ag, Pfizer, described in [11,14,21]) in QS-21 (Pfizer) or QS-21 alone. Two weeks after the final vaccination, animals underwent surgery, where a small incision was made in the thigh muscle parallel to the femur [22]. A stitch was placed in the deep tissue, then ~300 colony forming units (CFU) of *S. aureus* PFESA0158 in 10 µL phosphate-buffered saline (PBS) was instilled into the surgical site, which was then closed. Two days post-challenge, the mice were euthanized, quadriceps muscles were collected and homogenized, and serial dilutions of tissue homogenate were plated on tryptic soy agar (TSA; Becton Dickinson, Franklin Lakes, NJ, USA) plates (Becton Dickinson) to enumerate bacterial burden.

#### 2.2.2. Murine Bacteremia Model

Groups of 10 female (6–8–week–old) CD-1 mice (Charles River Laboratories, Wilmington, MA, USA) were vaccinated by subcutaneous injection at weeks 0, 3, and 6 with either vehicle or SA4Ag (Pfizer) in 23 µg AlPO_4_ as adjuvant. On week 8, the animals were challenged by intraperitoneal injection with ~4 × 10^8^ CFU of *S. aureus* CDC3 or PFESA0241. Three hours after challenge animals were exsanguinated by cardiac puncture and serial dilutions of blood plated on TSA to enumerate CFU.

#### 2.2.3. Murine Pyelonephritis Model

Groups of 5 female (6–8 week–old) CD-1 mice (Charles River Laboratories) were vaccinated by subcutaneous injection at weeks 0, 3, and 6 with either vehicle or SA4Ag (Pfizer, New York, NY, USA) in 23 µg AlPO_4_ as adjuvant. On week 8, the animals were challenged by intraperitoneal injection with ~2 × 10^8^ CFU of *S. aureus* Reynolds. Two days after challenge, kidneys were harvested and homogenized, and serial dilutions of homogenate plated on TSA to enumerate CFU.

#### 2.2.4. Rat Endocarditis Model

The vaccine was tested in two rat endocarditis models, a standard model [23] and a refined model that more accurately represents clinical disease.

##### Standard Model

Groups of female Sprague Dawley rats (5–6–week–old, 20/group, Charles River Laboratories) were vaccinated at weeks 0, 3, and 6, with SA4Ag in AlPO_4_ or with placebo. At week 8, two weeks after the final vaccination, catheter placement surgery was performed to generate sterile valvular vegetations [23]. Due to the complex nature of the model and surgery required, a proportion (<10%) of animals succumbed before infectious challenge due to the surgery and were thus not included in the analysis. Two days after surgery, animals were challenged intravenously with ~4 × 10^6^ CFU of *S. aureus* PFESA0158 in 0.1 mL PBS. Two days post-challenge, the rats were euthanized and hearts collected. Bacteria in homogenized heart tissue were enumerated (CFU/mL), and the arithmetic mean and 95% confidence interval (CI) were calculated for each treatment group.

##### Refined Model

In order to reflect the low challenge inoculum that has been reported in human infections [24], the endocarditis model was refined. Groups of female Sprague Dawley rats (5–6–week–old, 14/group; Charles River Laboratories) were vaccinated at weeks 0, 3, and 6, with SA4Ag in AlPO_4_ or with AlPO_4_ alone, and catheter placement surgery was conducted on week 8 as described above. Two days after surgery, animals were challenged intravenously with ~4 × 10^3^ CFU of *S. aureus* PFESA0186. Two days post-challenge, the rats were euthanized and hearts and kidneys collected. Tissues were plated and the presence or absence of bacterial CFU scored.

### 2.3. Assessment of Immune Responses to Vaccination

#### 2.3.1. Opsonophagocytic Activity Assay

Serologic responses to capsular polysaccharides CP5 and CP8 were measured by an opsonophagocytic activity (OPA) assay, as previously described [14]. Briefly, serial dilutions of heat-inactivated immune sera were mixed with either a CP5-expressing or CP8-expressing clinical isolate of *S. aureus* and allowed to opsonize the bacteria. The reaction mixtures were then mixed with baby rabbit complement (Pel-Freez, Rogers, AR, USA) and neutrophil-like HL-60 cells (ATCC^®^ CCL-240™, Manassas, VA, USA). An OPA antibody titer was defined as the reciprocal of the highest serum dilution resulting in 50% reduction of the number of bacterial colonies after incubation for 60 min at 37 °C when compared to the background control from which serum was omitted. Samples without detectable activity at the lowest serum dilution of 100 were assigned OPA titer values of 50.

#### 2.3.2. Competitive Luminex Immunoassay

Competitive Luminex-based immunoassays (cLIA) were used to quantify antigen-specific binding antibodies elicited by the investigational vaccine. The assays monitor the ability of each vaccine component to elicit antibodies that can compete with the binding of antigen-specific monoclonal antibodies (mAbs) that have shown functional activity either in vitro or in vivo. The ClfA mAb prevents binding of live *S. aureus* to fibrinogen [10], while the MntC mAb inhibits manganese uptake [25,26]. The mAbs used for the CP antigens facilitated killing of *S. aureus* as measured by the OPA assay. Spectrally distinct Luminex microspheres were coated individually with each of the antigens and incubated overnight with appropriately diluted serum samples. A mixture of phycoerythrin (PE)-labeled CP5-, CP8-, rmClfA-, and rP305A-specific mouse mAbs is then added to the microsphere/serum mixture, and after incubation, the unbound components are washed off. After reading in a Bio-Plex reader (Bio-Rad, Hercules, CA, USA), signals are converted to Units/mL.

## 3. Results

### 3.1. SA4Ag Is Immunogenic in Mice, Rats and Non-Human Primates

**Figure 1:**
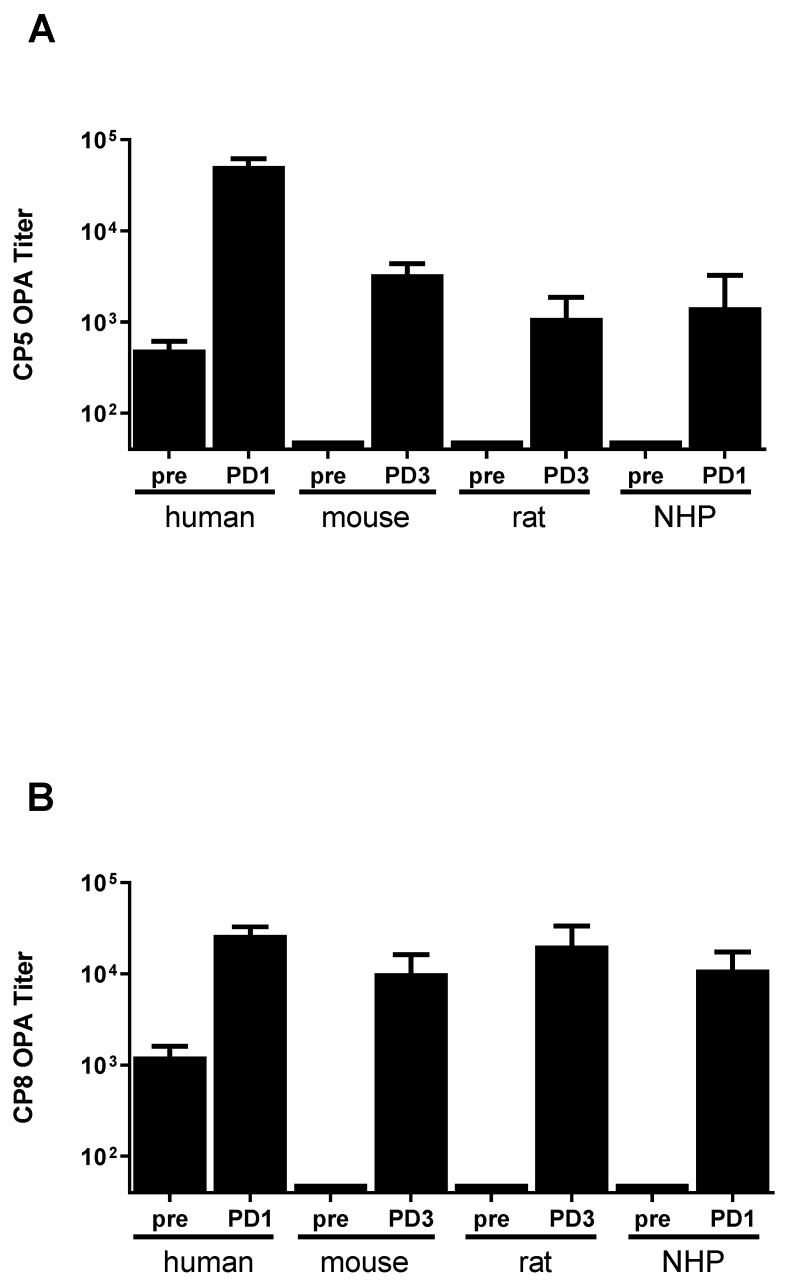

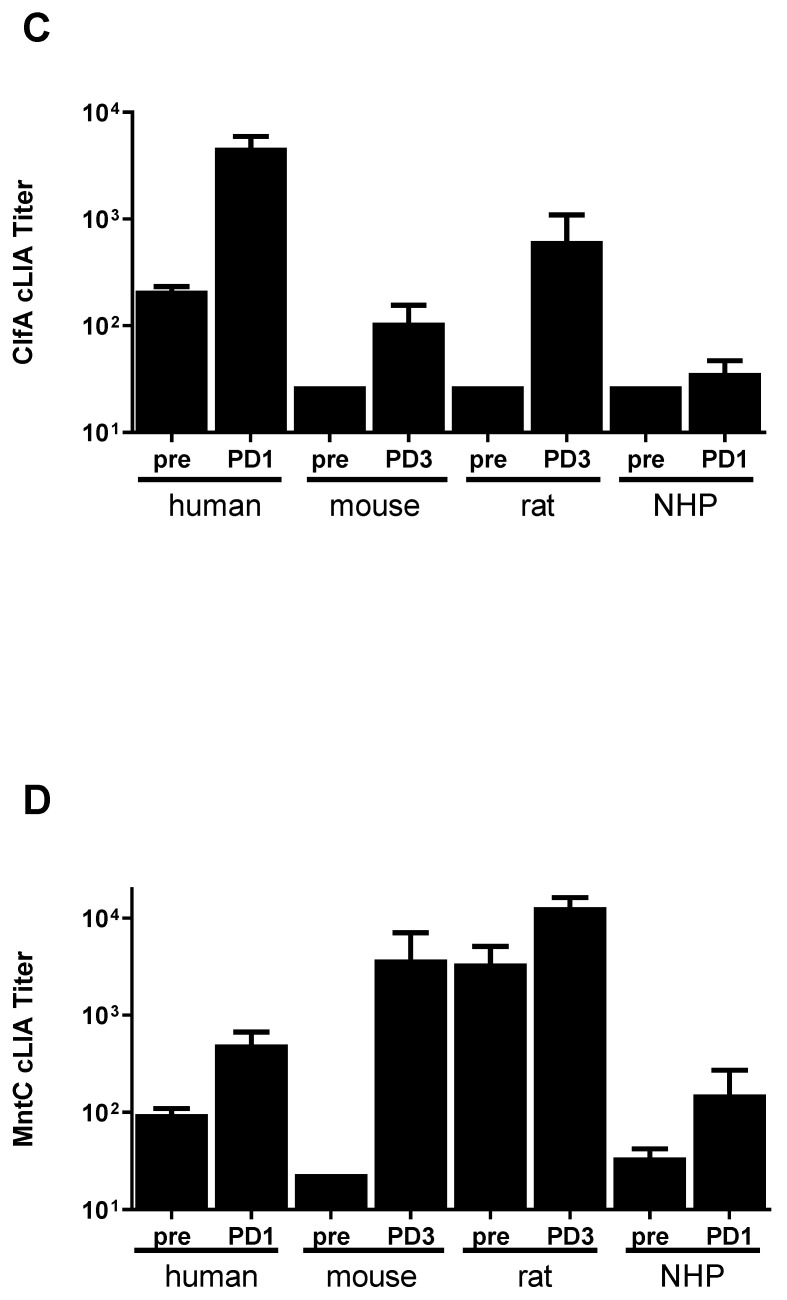
SA4Ag antigens are immunogenic in preclinical species. Immune responses against SA4Ag antigens CP5, CP8, ClfA, and MntC were measured before (pre) and after (PD) immunization. Rodents were immunized three times subcutaneously with SA4Ag + AlPO_4_ prior to sample collection post-dose 3 (PD3). Non-human primates (NHP) were immunized a single time with SA4Ag without adjuvant. Anti-capsular immune responses were measured by the OPA assay for CP5 (**A**) and CP8 (**B**). Anti-protein immune responses were measured by cLIA for ClfA (**C**) and MntC (**D**). Human responses to a single unadjuvanted dose of SA4Ag are included as a comparator, adapted from [27].

SA4Ag is comprised of capsular polysaccharides type 5 and type 8 CRM_197_-conjugates, ClfA, and MntC. SA4Ag was also shown to be able to elicit functional antibody responses in mice, rats and non-human primates, as measured by the OPA assay, which monitors the ability of serum samples to opsonize and induce uptake and killing of target bacteria by a neutrophil-like cell line, or by cLIA, which monitors the ability of serum to compete with mAbs for antigen binding. Importantly, the mAbs used in these assays inhibit the pathological function of the antigens they bind—e.g., fibrinogen binding in the case of ClfA [10] and manganese uptake in the case of MntC [26] (Figure 1). As Figure 1 shows, humans and non-human primates mount responses to the antigens in SA4Ag after a single dose (PD1), even in the absence of adjuvant, while rodents (mice and rats) require multiple immunizations with an adjuvant (e.g., AlPO_4_) to generate similar magnitude responses. This is likely due to the induction of an anamnestic response to the antigens in SA4Ag in non-human primates and humans, which is absent in the rodent species.

### 3.2. Immunization with SA4Ag Reduces Bacterial Burden in a Murine Model of Surgical Site Infection

Immunization with SA4Ag was evaluated in a murine model of surgical site infection. Analogous to human deep tissue surgical site infection, *S. aureus* was not introduced systemically but instead a low number of bacterial cells were inoculated into the surgical wound. As shown in Figure 1 and observed by others [28], mice respond relatively poorly to ClfA immunization, in comparison to both rats and humans, even in the presence of AlPO_4_ adjuvant. To enhance the ClfA response in a model where initial adhesion events are likely important, we considered the addition of an alternate, non–alum-based adjuvant. We limited our selection to adjuvants that are usable in human clinical trials, as some highly reactogenic adjuvants, such as Freund’s, overinflate immunogenicity. QS-21, a derivative of the bark of the *Quillaja saponaria* tree, has been used in human clinical trials and is purported to induce a more balanced IgG1/IgG2a response than alum-containing adjuvants in mice [29,30]. Addition of QS-21 to SA4Ag resulted in enhanced ClfA responses (geometric mean titer: 516.4; 95% CI: 222.4–1199.0; unpaired *t*-test with Welch’s correction: *p* = 0.048) as compared with AlPO_4_ (geometric mean titer: 42.8; 95% CI: 17.9–102.1). In the murine surgical site infection model, immunization with SA4Ag adjuvanted with QS-21 reduced bacterial burden (Table 2), illustrating an impact of SA4Ag in this difficult local infection model.

### 3.3. Immunization with SA4Ag Protects against MRSA Challenge in a Murine Bacteremia Model

*S. aureus* can disseminate from a local infection via the bloodstream. Therefore, SA4Ag was evaluated for its ability to reduce the bacterial burden in a murine bacteremia model, which mimics very early stages of hematogenous spread. Mice were immunized three times with SA4Ag with AlPO_4_ and then challenged with either *S. aureus* CDC3 or PFESA0241, both USA300 MRSA isolates. Blood was collected three hours post-challenge and bacteria were enumerated. Immunization with SA4Ag significantly reduced the number of recovered CFU with both USA300 MRSA isolates (Table 3).

### 3.4. Immunization with SA4Ag Reduces Bacterial Burden in a Murine Pyelonephritis Model

**Figure 2:**
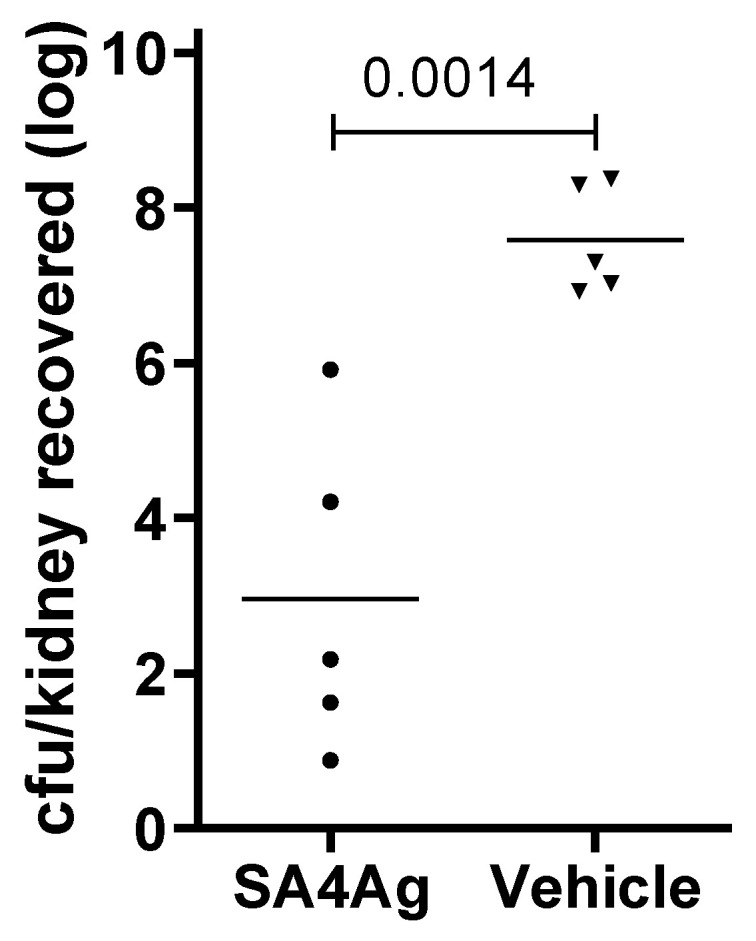
Immunization with SA4Ag reduces bacterial burden in a *S. aureus* pyelonephritis model. Female CD-1 mice (*n* = 5) were vaccinated at weeks 0, 3, and 6, with SA4Ag or with vehicle alone. Two weeks after the final vaccination animals were challenged with ~2 × 10^8^ *S. aureus* Reynolds. Two days post-challenge, the mice were euthanized and kidneys collected. Bacteria in kidneys were enumerated (colony-forming unit [CFU]/kidney). *p* value was calculated by Student’s *t*-test.

*S. aureus* can cause infection at sites distant from the initial site of infection, and the kidney is a common end organ for *S. aureus* infection. A murine pyelonephritis model was used to evaluate the ability of SA4Ag to reduce the bacterial burden in the kidney. Mice were immunized with SA4Ag and then challenged with *S. aureus* Reynolds. Two days after challenge, kidneys were harvested and homogenized to enumerate bacterial burden. Immunization with SA4Ag significantly reduced the number of recovered CFU from kidneys (Figure 2).

### 3.5. Immunization with SA4Ag Protects against Both CP5- and CP8-Expressing S. aureus in a Rat Endocarditis Model

*S. aureus* can cause infection at sites distant from the initial site of infection. SA4Ag was evaluated for its ability to reduce the bacterial burden in a model of foreign body–like infection, i.e., rat endocarditis following catheterization, considered a very stringent preclinical model. A catheter was inserted into the heart via the jugular vein across the aortic valve to create sterile valvular vegetations. Two days after catheter placement, animals were challenged intravenously with *S. aureus*. In two out of three studies, immunization with SA4Ag reduced the bacterial burden of a CP5-expressing *S. aureus* clinical isolate from infected heart tissue (Table 4). Meta-analysis of these three studies demonstrated a significant reduction in recovered CFU (Table 5, *p* = 0.0126).

The endocarditis model was next refined to improve clinical relevance by reducing the challenge inoculum. It is believed that *S. aureus*-induced foreign body infections and endocarditis are caused by the seeding of very low numbers of bacteria introduced to the bloodstream either during surgery or as a result of minor tissue injury, such as resulting from a scratch or brushing of teeth [24]. To model human bacterial exposure more closely, the infectious inoculum was reduced to a level just above the threshold for achieving an infection. In this model, *S. aureus* preferentially seeds the damaged valvular tissue and the kidneys; no bacterial burden was detected in the liver, spleen or lungs. In the refined model, the complete absence of detectable infection in both hearts and kidneys following immunization with SA4Ag was evaluated. Rats were immunized subcutaneously three times on weeks 0, 3, and 6 with SA4Ag, and then underwent catheter placement surgery. Two days after catheter placement, rats were challenged intravenously with a CP8-expressing clinical *S. aureus* isolate, PFESA0186. Immunization with SA4Ag reduced the number of animals with detectable *S. aureus* infection in the hearts and kidneys in two separate experiments. Meta-analysis of the two experiments showed a significant reduction in the number of infected animals upon immunization, representing a vaccine efficacy of 88.7% (Table 5).

## 4. Discussion

A four-antigen *S. aureus* vaccine (SA4Ag) was designed as a candidate to prevent invasive *S. aureus* disease in postsurgical populations. Therefore, SA4Ag was evaluated for the ability to protect against invasive *S. aureus* disease in a series of preclinical models that mimic aspects of human postsurgical infection. Individual components of SA4Ag have previously been shown to elicit robust antibody responses and efficacy in rodents [11,14,21,31], but efficacy of the combined SA4Ag vaccine in multiple preclinical models has not previously been reported. SA4Ag was found to significantly reduce or abrogate infection in murine models of surgical site infection, bacteremia, and in two iterations of a rat endocarditis model. These models mimic the progression of *S. aureus* postsurgical invasive disease from a local deep tissue infection, through hematogenous spread, and dissemination to distant tissue sites, such as the heart valves and kidneys. Together, the responses seen in these models demonstrated that vaccination with SA4Ag elicits an immune response that restrains multiple stages of invasive preclinical *S. aureus* infection and suggested that similar positive outcomes could be achieved in the clinic. Importantly, in the refined rat endocarditis model, a complete absence of infection was observed in 96% of vaccinated animals, reflecting a vaccine efficacy of 88.7%. This is the first time to our knowledge that a sterilizing immune response has been demonstrated after administration of an *S. aureus* vaccine in a preclinical model, and supported moving the SA4Ag into clinical development.

*S. aureus* disease is challenging to effectively model in animals due to the organism’s adaptation to the human host environment and the lack of a defined correlate of protection for invasive *S. aureus* disease. As demonstrated in Figure 1, rodents, which are commonly used in preclinical models of *S. aureus* pathogenesis, required three doses of SA4Ag with an adjuvant to achieve immune responses similar to those seen in non-human primates and humans after a single unadjuvanted dose of SA4Ag. It is possible, therefore, that the response of repeatedly immunized naïve animals does not fully recapitulate the maturation of an immune response originally elicited through natural exposure. The response to ClfA may serve as a case in point. Naïve mice respond poorly to ClfA, even after multiple immunizations in the presence of adjuvant, while humans and non-human primates respond well after a single dose. Interestingly, even though humans respond well to a single dose of ClfA-containing SA4Ag, implying ClfA is eliciting an anamnestic response, a functional anti-ClfA response, which can block the binding of ClfA to fibrinogen, is only observed after immunization [10]. This highlights the need to match the right vaccine with antigen presentation in the correct format.

Invasive *S. aureus* infection can proceed from a local inoculation site, through dissemination in the bloodstream, to distant tissues. A given vaccine candidate may show efficacy in one or more models, but it is the combination of vaccine candidates and evaluation in relevant models of infection that improves the robustness of the conclusions drawn from the preclinical program. Based in part on the preclinical data included here, the *S. aureus* vaccine was advanced into clinical testing. Prior to the clinical assessment of SA4Ag, two other *S. aureus* vaccines also failed to demonstrate efficacy in the clinic, despite having supportive preclinical data. Both vaccines had a single antigenic target, either capsular polysaccharide or IsdB. The StaphVax vaccine, comprised of capsular polysaccharides type 5 and type 8 conjugated to Pseudomonas exotoxin A, did not meet its Phase III clinical endpoint. It has been suggested that this may have resulted from quality variations of the conjugate used in the trial, as opposed to a failure of the mechanism of action [32]. Thus, *S. aureus* capsular polysaccharides had remained an attractive vaccine candidate but potentially not sufficient to protect against invasive *S. aureus* disease. A second single-antigen *S. aureus* vaccine, V710, contained the iron uptake component, IsdB, and also did not meet its primary efficacy endpoint (prevention of serious postoperative *S. aureus* infections following cardiothoracic surgery) in a randomized Phase 2b/3 trial. Additionally, V710 was associated with increased mortality among patients who developed *S. aureus* infections [33]. IsdB did protect mice in preclinical lethal challenge models [17,18]. However, *S. aureus* has multiple redundant iron acquisition systems and prefers human hemoglobin as an iron source, which may have limited the predictive value of the models used to evaluate this antigen [34]. In preclinical models, the protection seen with IsdB was improved with incorporation of additional *S. aureus* antigens [35]. The recognition that a single antigen approach may not be sufficient to protect against *S. aureus*-mediated disease spurred the development of several multi-antigen approaches in addition to SA4Ag [36,37,38].

In an early Phase 1/2 clinical study (NCT01364571), SA4Ag elicited robust functional immune responses and showed an acceptable safety profile [27]. However, a large randomized, placebo-controlled Phase 2b study (STRIVE; NCT02388165) in adult patients undergoing elective open posterior spinal fusion surgery [39], the results of a pre-planned interim analysis indicated low statistical probability to meet the pre-defined primary efficacy objective—prevention of invasive *S. aureus* disease (bloodstream and surgical site infections), leading to the decision to terminate the study and discontinue the clinical development [40]. This failure indicates that despite protection seen in the current preclinical studies, the animal models that were used had limitations that did not enable prediction of clinical efficacy of the vaccine. One such potential shortcoming is the measure of protection. For example, the absence of detectable infection was used as the criterion of induced protection for the refined endocarditis model. A second shortcoming is that the infection models all evaluated early time points before any animals could have succumbed to infection. Examination at later time points might have determined any impact of the vaccine on survival, and changes in pathology could be identified (e.g., wound size and induration, paralysis or some other signs of morbidity, or histological changes). Examination of the immune responses elicited by the vaccine in more detail (innate vs. adaptive) might also have provided more insight into the mechanism(s) of protection.

Since there is no human correlate of protection against *S. aureus* infection, establishing reliable animal models for preclinical evaluation of *S. aureus* vaccine candidates remains very challenging [19,20]. This is further supported by the observation that while non-human primates required a single dose of SA4Ag without adjuvant to generate functional immune responses, rodents required multiple doses of SA4Ag with adjuvant to achieve similar levels of immune response. Therefore, current preclinical efficacy models play an important yet circumscribed role in illustrating the protective potential of a *S. aureus* vaccine candidate. Animal models are useful in this context for demonstrating proof of principle or proof of mechanism, but ultimately clinical efficacy data, obtained using carefully selected vaccine formulations and target populations, is required until a reliable correlate of protection is identified [19,20]. Identification of a relevant correlate of protection for invasive *S. aureus* disease and development of preclinical models that accurately reflect these human correlates is needed to improve effective preclinical evaluation of *S. aureus* vaccine candidates.

## 5. Conclusions

Although a *S. aureus* vaccine candidate, SA4Ag, showed promise in the various preclinical models presented, it ultimately did not show clinical efficacy. SA4Ag is the latest in a series of *S. aureus* vaccines that showed protection in preclinical models, which did not translate into positive clinical outcomes. This highlights the need to identify relevant correlates of protection for invasive *S. aureus* infection, and to develop appropriate preclinical models that better predict clinical efficacy.

## Figures and Tables

**Table 1:** *S. aureus* Strains Used in These Studies.

Strain Name	Capsule Type	Source	MRSA/MSSA	ST/CC	Comments
CDC3 (USA300)	5	ID	MRSA	ST8/CC8	PVL+
PFESA0241 (USA300)	5	ID	MRSA	ST8/CC8	Mec IV, PVL+ TSST-neg
PFESA0158	5	ID	MSSA	ST28/CC25	
PFESA0186	8	Carriage	MSSA	ST57/CC30	
Reynolds	5	ID	MSSA	ST25/CC25	

ID, Invasive Disease; MRSA, Methicillin-resistant *Staphylococcus aureus*; MSSA, Methicillin-sensitive *Staphylococcus aureus*; PVL+, Panton-Valentine leukocidin-positive; ST/CC, sequence type/clonal complex; TSST-neg, Toxic shock syndrome toxin-negative.

**Table 2:** Immunization with SA4Ag Reduces *S. aureus* Burden in a Murine Surgical Site Infection Model.

Trial	Vaccine	Number of Animals	Mean log CFU/mL (95% CI)	*p* Value vs. Control
1	SA4Ag	20	4.65 (3.90–5.40)	0.0393
	Vehicle	20	5.72 (4.99–6.45)	
2	SA4Ag	15	6.42 (5.07–7.77)	0.0330
	Vehicle	13	7.89 (6.95–8.83)	
3	SA4Ag	17	5.95 (4.60–7.31)	0.2899
	Vehicle	16	6.45 (5.11–7.81)	
Meta-analysis	SA4Ag	48	6.29 (5.57–7.01)	0.0126
	Vehicle	43	7.36 (6.75–7.97)	

Female CD1 mice (*n* = 10–20) were immunized on weeks 0, 3, and 6 with SA4Ag + QS-21 or QS-21 alone. On week 8, animals underwent surgery by placing an incision and stitch into the quadriceps muscle. Approximately 300 colony-forming units (CFU) of *S. aureus* PFESA0158 were instilled into the surgical site. Two days after surgery, tissue was harvested and bacterial burden was enumerated. *p* values were determined by Student’s *t*-test.

**Table 3:** Immunization with SA4Ag Reduces Bacterial Burden in a *S. aureus* Bacteremia Model.

Challenge	Trial	Vaccine	Number of Animals	Mean log CFU/mL (95% CI)	*p* Value vs. Control
*S. aureus* CDC3 (USA300)	1	SA4Ag	10	4.65 (4.33–4.97)	0.0056
		Vehicle	10	5.35 (4.96–5.73)	
	2	SA4Ag	10	4.49 (4.07–4.90)	0.0147
		Vehicle	10	5.24 (4.76–5.72)	
	3	SA4Ag	10	3.63 (3.25–4.01)	0.0056
		Vehicle	10	4.47 (4.00–4.94)	
	Meta-analysis	SA4Ag	30	4.25 (4.00–4.51)	<0.0001
		Vehicle	30	5.02 (4.75–5.29)	
*S. aureus* PFESA0241 (USA300)	1	SA4Ag	10	4.17 (3.83–4.51)	0.0491
		Vehicle	10	4.68 (4.25–5.12)	
	2	SA4Ag	10	4.26 (3.93–4.59)	0.0157
		Vehicle	10	4.82 (4.48–5.15)	
	3	SA4Ag	10	4.38 (3.98–4.78)	0.0455
		Vehicle	10	4.88 (4.54–5.22)	
	Meta-analysis	SA4Ag	30	4.27 (4.09–4.45)	0.0002
		vehicle	30	4.79 (4.60–4.98)	

Female CD-1 mice (*n* = 10) were vaccinated at weeks 0, 3, and 6, with SA4Ag or with vehicle alone. Two weeks after the final vaccination animals were challenged with ~2 × 10^8^ *S. aureus* CDC3 or PFESA0241. Three hours post-challenge, the mice were euthanized and blood collected. Bacteria in blood were enumerated (colony-forming unit [CFU]/mL), and the log CFU reduction with 95% confidence interval (CI) was calculated compared to the vehicle-treated control. *p* values were determined by Student’s *t*-test.

**Table 4:** Immunization with SA4Ag Reduces Recovered CFU of a CP5-expressing *S. aureus* Clinical Isolate in a Rat Endocarditis Model.

Trial	Vaccine	Number of Animals	Mean Log CFU/mL (95% CI)	*p* Value vs. Control
1	SA4Ag	16	6.52 (5.20–7.84)	0.0319
	Vehicle	14	7.88 (7.19–8.58)	
2	SA4Ag	15	6.42 (5.07–7.77)	0.0330
	Vehicle	13	7.89 (6.95–8.83)	
3	SA4Ag	17	5.95 (4.60–7.31)	0.2899
	Vehicle	16	6.45 (5.11–7.81)	
Meta-analysis	SA4Ag	48	6.29 (5.57–7.01)	0.0126
	Vehicle	43	7.36 (6.75–7.97)	

Groups of female Sprague Dawley rats (20/group) were vaccinated at weeks 0, 3, and 6, with SA4Ag in AlPO_4_ or with AlPO_4_ alone. Two weeks after the final vaccination, catheter placement surgery was performed to generate sterile valvular vegetations. A certain number of animals succumbed due to the surgery and were not included in the analysis. Two days after surgery, animals were challenged intravenously with *S. aureus* PFESA0158. Two days post-challenge, the rats were euthanized and hearts collected. Bacterial burden in heart tissue was enumerated (colony-forming unit [CFU]/mL) and the arithmetic mean and 95% confidence interval (CI) were calculated for each treatment group.

**Table 5:** Immunization with SA4Ag Reduces the Number of *S. aureus* Infections in a Rat Endocarditis Model of Infection.

Experiment	Vaccine	Number of Infected Animals	Number of Uninfected Animals	*p* Value vs. Control
1	SA4Ag	0	11	0.0983
	Vehicle	4	9	
2	SA4Ag	1	11	0.0730
	Vehicle	6	7	
Meta-analysis	SA4Ag	1	22	<0.0001
	Vehicle	10	16	

Female Sprague Dawley rats (*n* = 11–14) were vaccinated at weeks 0, 3, and 6, with SA4Ag in AlPO_4_ or with AlPO_4_ alone. Two weeks after the final vaccination, catheter placement surgery was performed to generate sterile valvular vegetations. A certain number of animals succumb due to the surgery and were not included in the analysis. Two days after surgery, animals were challenged intravenously with ~4 × 10^3^ colony-forming unit (CFU) *S. aureus* PFESA0186, a CP8-expressing clinical isolate. Two days post-challenge, the rats were euthanized, and tissue bacterial burden was quantitated in hearts and kidneys.

## Data Availability

The data presented in this study are available on request from the corresponding author.
